# Morphological observation and protein expression of fertile and abortive ovules in *Castanea mollissima*

**DOI:** 10.7717/peerj.11756

**Published:** 2021-07-21

**Authors:** Bingshuai Du, Qing Zhang, Qingqin Cao, Yu Xing, Ling Qin, Kefeng Fang

**Affiliations:** 1 Beijing Advanced Innovation Center for Tree Breeding by Molecular Design, Beijing University of Agriculture, Beijing, China; 2 College of Landscape Architecture, Beijing University of Agriculture, Beijing, China; 3 Key Laboratory for Agricultural Application and New Technique, College of Plant Science and Technology, Beijing University of Agriculture, Beijing, China; 4 Key Laboratory of Urban Agriculture (North China, Ministry of Agriculture P. R. China), Beijing University of Agriculture, Beijing, China

**Keywords:** *Castanea mollissima* Blume, Fertile ovules, Abortive ovules, iTRAQ

## Abstract

Chinese chestnuts (*Castanea mollissima* Blume.) contain 12–18 ovules in one ovary, but only one ovule develops into a seed, indicating a high ovule abortion rate. In this study, the Chinese chestnut ‘Huaihuang’ was used to explore the possible mechanisms of ovule abortion with respect to morphology and proteomics. The morphology and microstructure of abortive ovules were found to be considerably different from those of fertile ovules at 20 days after anthesis (20 DAA). The fertile ovules had completely formed tissues, such as the embryo sac, embryo and endosperm. By contrast, in the abortive ovules, there were no embryo sacs, and wide spaces between the integuments were observed, with few nucelli. Fluorescence labelling of the nuclei and transmission electron microscopy (TEM) observations showed that cells of abortive ovules were abnormally shaped and had thickened cell walls, folded cell membranes, condensed cytoplasm, ruptured nuclear membranes, degraded nucleoli and reduced mitochondria. The iTRAQ (isobaric tag for relative and absolute quantitation) results showed that in the abortive ovules, low levels of soluble protein with small molecular weights were found, and most of differently expressed proteins (DEPs) were related to protein synthesis, accumulation of active oxygen free radical, energy synthesis and so on. These DEPs might be associated with abnormal ovules formation.

## Introduction

Chinese chestnut (*Castanea mollissima* Blume) is an important crop/tree species and known as a ‘wood cereal-and-oil-yielding’ species and an ‘iron crop’ in China (Jiang, Liang & Tian, 2020). Chestnut kernels are rich in carbohydrates and protein but do not contain gluten or cholesterol, which considered as a healthy food (Chang et al., 2020). Besides, chestnut trees are planted on mountainous areas and are income sources for people in China. (Du et al., 2020). Chestnut is a monoecious deciduous tree. Its staminate flowers have 2–3 catkins (sometimes more). Female flowers are fascicled at the bottom of mixed inflorescence (Chen et al., 2019). There are 12–18 ovules in each pistil, but only one typically develops into a seed. The rest are aborted, but the exact cause of the abortion is not clear.

Ovules are the female reproductive tissues and provide mechanical support and nutrition for the formation and development of the female gametophyte, thus playing a major role in sexual reproduction (Yang et al., 2016). During the double fertilization of angiosperm, a pair of sperms are released from pollen tube, one fuses with the haploid egg cell, generating a diploid embryo, and the other fuses with the diploid central cell, forming the triploid endosperm (Hamamura et al., 2011; Leshem et al., 2012). All tissues within the ovule follow a directed and highly ordered pattern of division, and they eventually develop into mature seeds under the regulation of orderly and selective gene expression. In this process, the developmental retardation of any stage can result in ovule abortion. Awasthi et al. (2012) suggested that the ovule phenotypes of sterile *Oryza sativa* L. lines varied and megaspore division was abnormal. Yang suggested that megaspore degradation was the major cause of abortive ovules (Yang et al., 2016). For *Armeniaca vulgaris* Lam., the abnormal metabolism of reactive oxygen species leads to abortive ovules (Liu et al., 2012). Previous research has also shown significant differences in the microstructure of fertile ovules and abortive ovules. Robinson-Beers, Pruitt & Gasser (1992) indicated that sterile ovules have only a single perianth, and no functionally mature embryo sac could be formed within the ovules. Wetzsteini et al. (2013) reported an obvious gap between the inner integument and the outer integument during the development of abortive ovules in *Punica granatum*. Sun et al. (2016) investigated the causes of abortion during ovule development in apricot, and found that in the abortive ovules, approximately half of the embryo sac did not differentiate or disintegrated when the plant was pollinated and fertilized, while in the other half the megaspore mother cells were stagnated at various stages in meiosis. Tang & Kato (2002) investigated the causes of ovule abortion in rice, which showed that the abortion happened on the formation of the functional megaspore, and both of the three nonfunctional megaspores near the micropyle and the functional megaspore farthest from the micropylar end were degenerated in the abortive ovules.

The concept of proteomics was first proposed by Marc Wilkins. Proteomics refers to the sum of all proteins expressed by a cell or tissue in a given time frame and specific environment (Wilkins et al., 1996). Proteomic approaches have recently been widely applied to the study of plant genetics and fruit development. With the continuous advancement of quantitative proteomics and the development of experimental techniques, quantitative proteomics has gradually become an area of active research, and iTRAQ (Isobaric tag for relative and absolute quantitation) has become a commonly used method (Wang et al., 2016; Qin et al., 2016). Previous research has shown that in the development of many plants, such as longan, grapes, and corn, there are significant differences in protein content in different developmental stages, and the soluble protein content of abortive seeds is much lower than that of normal seeds (Wasinger et al., 1995; Liu et al., 2010; Cagnola et al., 2018; Zhu et al., 2019). By analysing the different proteins contained in fertile pollen and abortive pollen of wheat, Geng et al. (2018) found that the downregulation of enzymes related to energy metabolism caused pollen abortion in wheat, such as glyceraldehyde-3-phosphate dehydrogenase in the glycolysis pathway, isocitrate dehydrogenase and citrate synthase in the tricarboxylic acid cycle and adenosine-triphosphate (ATP) synthases in the oxidative phosphorylation pathway. Zhang et al. (2017) identified 4,192 proteins expressed differentially between fertile and sterile ovules, and most of the differential expressed proteins participated in DNA replication, active oxygen metabolism, ATP synthesis and so on. In addition, previous research had shown that abnormal metabolism of reactive oxygen species could also affect fertility of plants (Zhang et al., 2014; Cheng et al., 2018; Nie, Cheng & Hua, 2020). There are relatively few studies on ovule abortion in the nuts of the Chinese chestnut tree. Therefore, this study aimed to explore the possible mechanism of ovule abortion by combining structural observations and proteomics.

## Materials & methods

### Plant material

The anthesis of *C. mollissima* cv. ‘Huaihuang’ begins at mid-June. One hundred burrs from 20 chestnut trees were monitored at 20 days after anthesis (DAA) from the Chestnut Experiment Station in Huairou District, Beijing, China. At this time, fertile and abortive ovules in the same ovary could just be distinguished by the naked eyes.

### Paraffin sections

Morphological structure of ovaries and ovules were observed according to the method described by Du et al. (2020). The ovaries and ovules were dehydrated in a graded series of ethanol (50%, 70%, 80%, 95% and 100%) followed by a xylene/ethanol series (ethanol/xylene 1:1 and 100% xylene) and wax/xylene series (wax/xylene 1:1 and 100% wax), and according to the size of samples, the timing of dehydration and the wax-impregnated were adjusted appropriately; Samples and the wax solution were poured into a stacked carton, the position of the ovaries and ovules were quickly adjusted before the wax solution could solidify. Paraffin-embedded samples were cut into slices of 7-μm thickness using a rotary microtome (Leica RM 2235) and dried on a hot plate at 45 °C overnight; The next day, the tissue biopsies were deparaffinized by a xylene/ethanol series (100% xylene, and ethanol/xylene 1:1) followed by an ethanol series (100%, 90%, 80%, 70%, 60% and 50%), then were hydrated in reverse.

### Fluorescent labeling of cell nuclei

Propidium iodide (PI; Sigma, Kawasaki, Kanagawa, Japan) powder was dissolved at 30 ng/ml in PBS buffer. Then the tissue sections were incubated for 10–15 min in the PI stain (triplicates). DAPI (4′,6-diamidino-2-phenylindole; Sigma, Kawasaki, Kanagawa, Japan) powder was dissolved at 1 μg/ml in PBS buffer, and the tissue sections were incubated for 5–10 min in the DAPI stain (triplicates). After staining, the tissue biopsies were observed under a fluorescence microscope (Olympus BX51, Japan), and the images were captured with a CCD color camera.

### Transmission electron microscopy

(1) The samples were fixed in 2.5% glutaraldehyde and then placed under vacuum for 2.5 h until the ovules were completely immersed in the fixative, then the tubes filled with the sample material were placed at 4 °C for overnight fixation. (2) After fixation, the ovules were washed 10 times for 10 min each with phosphate buffer (10 mM, pH 7.2). (3) Osmium acid was mixed with PBS in equal volumes in a centrifuge tube, and the sample was fully immersed in the mixture for 1.5 h with vibration. After osmic acid fixation was complete, the fixed ovules were washed 10 times for 10 min each with phosphate buffer. (4) After rinsing, the ovules were removed to fresh centrifuge tubes using a toothpick, then the fixed samples were dehydrated in a graded series of ethanol (15 min per step) at room temperature, followed by an acetone wash. (5) Acetone and a resin embedding agent were mixed at ratios of 1:1, 1:2 and 1:3. The ovules were impregnated separately for 8 h in the mixtures. (6) The samples were embedded at both ends of the embedding plate by the resin. (7) The embedding material was polymerized at 60 °C for 15 days. (8) The embedding block was cut into 50 nm slices by an ultra-thin slicing technique; the slices were then placed on a copper mesh. (9) The sections of the copper mesh were double-stained with 2% uranium acetate and 0.5% lead acetate. (10) The copper mesh was observed under a transmission electron microscope.

### Protein digestion and iTRAQ labeling of samples

iTRAQ was used to identify differences in protein expression between fertile ovules and abortive ovules in early Chinese chestnut ovaries. This experiment was conducted at the Beijing Protein Innovation Co., Ltd. Three replications were carried out. The experimental procedures included: (1) Protein extraction. Triplicates of fertile ovules and abortive ovules were homogenized in precooled extraction buffer composed of 50 mM Tris-HCl, 2 mM EDTA, 100 mM KCl, 700 mM sucrose solutions and Phenol Tris. Following this step, the mixture was fully oscillated and centrifuged at 15,000×*g* for 10 min, then the supernatant obtained was mixed with ammonium acetate and acetone. Subsequently, the mixture was centrifuged at 15,000×*g* for 10 min, then the sediment obtained was mixed with lysis buffer (8 M Urea, 30 mM HEPES, 1 mM PMSF, 2 mM EDTA and 10 mM DTT). Then supernatant was precipitated with acetone again for 3 hours, the addition of redissolve buffer composed with 50% TEAB and 0.1% SDS to the sediment by centrifugation at 20,000×*g* for 30 min to get supernatant. Finally, the peptides were quantified by Bradford method. (2) Protein digestion. The peptides were digested overnight with 1 μg/μl trypsin in 100 μl TEAB (1 M) at 37 °C, which were freeze-dried and then redissolved with 30 μl TEAB solution (TEAB/water 1:1) for iTRAQ labelling. (3) Peptide marking. Peptide samples were divided into three batches and labeled with labeling reagents. For one batch, fertile ovules were labeled with 113, 114 and 115, for another batch, abortive ovules were labeled with 116, 117 and 118. Samples in each batch were mixed after labeled and then subjected to MS/MS analysis. (4) LC-MS/MS analysis. After desalted, the labeled peptides were separated and determined for the MS analysis by HPLC (High Performance Liquid Chromatography). This step was performed *via* a “trap and elute” configuration and the mobile phase included A (25% ACN, 10 mM KH_2_PO_4_) and B (25% ACN, 2 M KCL, 10 mM KH_2_PO_4_). The peptides were separated over 45 min by a gradient of 5–80% of mobile phase B at a flow rate of 400 nl/min combined with a Q Exactive mass spectrometer and the chromatographic column of model C18 was choice with a particle size of 5 μm. Then to evaluate the performance of this mass spectrometry on the iTRAQ labeled samples, two MS/MS acquisition modes, higher collision energy dissociation (HCD) was employed. And to optimize the MS/MS acquisition efficiency of HCD, normalized collision energy (NCE) was systemically examined 28, stepped 20%. (5) Qualitative and quantitative analysis of the proteins. Mass spectrograms were filtered by using Proteome Discoverer software v.1.3 and researched with mascot, searching parameters were defined as following: fixed modification-carbamidomethyl; sample style-iTRAQ 8 plex; peptide tol-15 ppm; MS/MS tol-20 mmu; digestion-trypsin; database-uni_rosids_71275. Then the search results and the spectrograms after the first step were quantitatively analyzed, quantitative analysis parameters were defined as following: protein ratio type-median; minimum peptides-1; normalisation method-median. In addition, the significant differences and the SD value of protein concentrations, total protein and DEPs expression ratio from fertile and abortive ovules were analyzed and calculated by SPSS software.

### Bioinformatics analysis of differential proteins

The differently expressed proteins (DEPs) were defined with fold-change ratios of log2 > 1.2 or < 0.8. Analysis of DEPs were carried as previously described (Geng et al., 2018). In a short, additional filters were initially conducted to screen out proteins with *p*-value < 0.05 (95% confidence) and ion score or expected cutoff < 0.05 (95% confidence). These screened DEPs were subsequently characterized by Blast2GO and the non-redundant protein database. The significance of the GO analysis was further confirmed in the KEGG database, in which each DEP was classified into a specific pathway. Meanwhile, the mass spectrometry proteomics data have been deposited to the ProteomeXchange Consortium *via* the PRIDE partner repository with the dataset identifier PXD021317.

### Western blot

This experiment was carried out at the Beijing Protein Innovation Co., Ltd., three replicates were performed. In this experiment, one peptide sequence containing the specific amino acids corresponding to the mature glyceraldehyde-3-phosphate dehydrogenase (GAPDH) sequence, was obtained by chemical synthesis. Synthetic peptide was purified by high-performance liquid chromatography and coupled with keyhole limpet hemocyanin. Then GAPDH was detected by western blotting, with steps below. First, filter paper, a PVDF membrane and an acrylamide gel were placed in the film transfer slot with transfer solution, and 100 V was applied for 2 hours to transfer the proteins to the PVDF membrane. The PVDF membrane was then placed in a primary antibody solution, followed by washing to remove non-specific binding. The membrane was incubated with horseradish peroxidase (HRP)-conjugated goat anti-rabbit immunoglobulin G at room temperature for 1–2 h and then incubated in ECL2 reagent for 5 min in the dark. The PVDF membrane was then placed in a development folder, air bubbles were rolled out. The final color was developed with a solution containing 3,3-diaminobenzidine tetrahydrochloride as HRP substrate.

## Results

### Morphologies of Chinese chestnut burrs, ovaries and ovules

**Figure 1:**
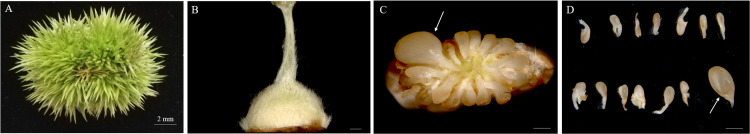
Morphologies of Chinese chestnut burrs, ovaries and ovules. (A) The morphological observation of Chinese chestnut shell of 20 DAA, the chestnut spines had hardened during this period; (B) ovary of 20 DAA; (C, D) ovules of 20 DAA, the fertile ovules volume greater than the abortive ovules; the arrow points to fertile ovule, DAA, Days after anthesis, Bar = 500 μm in B–D.

The chestnut anthesis begins in mid-June. Female burrs were collected approximately 20 DAA. During this period, the chestnut spines become markedly stiffened and the ovary becomes enlarged (Figs. 1A, 1B). Fertile and abortive ovules in the same ovary could be distinguished by eyes. Based on the anatomy of the ovary, there were 12–18 ovules per ovary, and ovules were localized at the apex of the ovary connected to the style. Other ovules were aborted except the fertile ovule that could develop normally (Figs. 1C, 1D).

### Microstructure of fertile and abortive ovules

**Figure 2:**
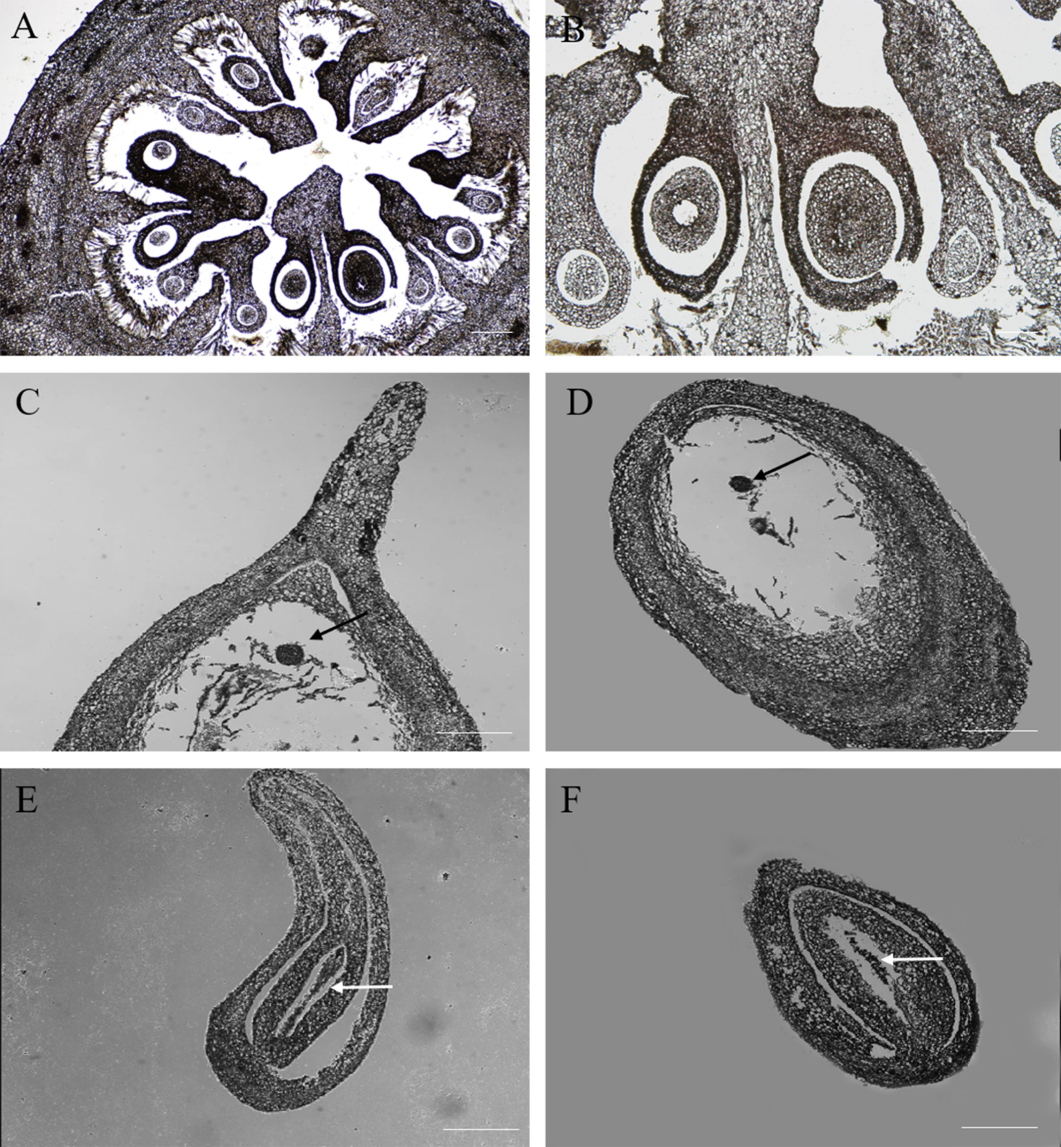
The microstructure observation of fertile and abortive ovules at 20 days after anthesis. (A, B) the cross section of ovary, the ovary consisted of nine locules, and each locule had two inverted ovules; (C, D) The longitudinal section of fertile ovule, the black arrow points to embryo, and embryo developed into a sphere; (E, F) the longitudinal section of abortive ovule, the white arrow points to nucellar tissue; Bar = 100 μm.

Cross sections of the ovaries revealed 6–9 ventricles per ovary, and each ventricle had two different sizes of anatropous ovules; it is possible that the smaller one might be aborted earlier (Figs. 2A, 2B). The longitudinal section of the fertile ovules showed that they contained completely formed tissues, such as the embryo sac, embryo and endosperm, and the structures could support the normal processes of pollination, fertilization and embryogenesis. At this time (20 DAA), we could also see that the embryo developed into a sphere near the end of the funicle, and the inner integument was closely connected to the outer integument (Figs. 2C, 2D). But in the abortive ovules, there were some key parts missing, including embryo sac, embryo and endosperm; and obvious gap was present between the inner and outer integument compared with fertile ovules; furthermore, remnants of nucellus could still be seen in the abortive ovules (Figs. 2E, 2F).

### Nuclei of fertile and abortive ovules cells

**Figure 3:**
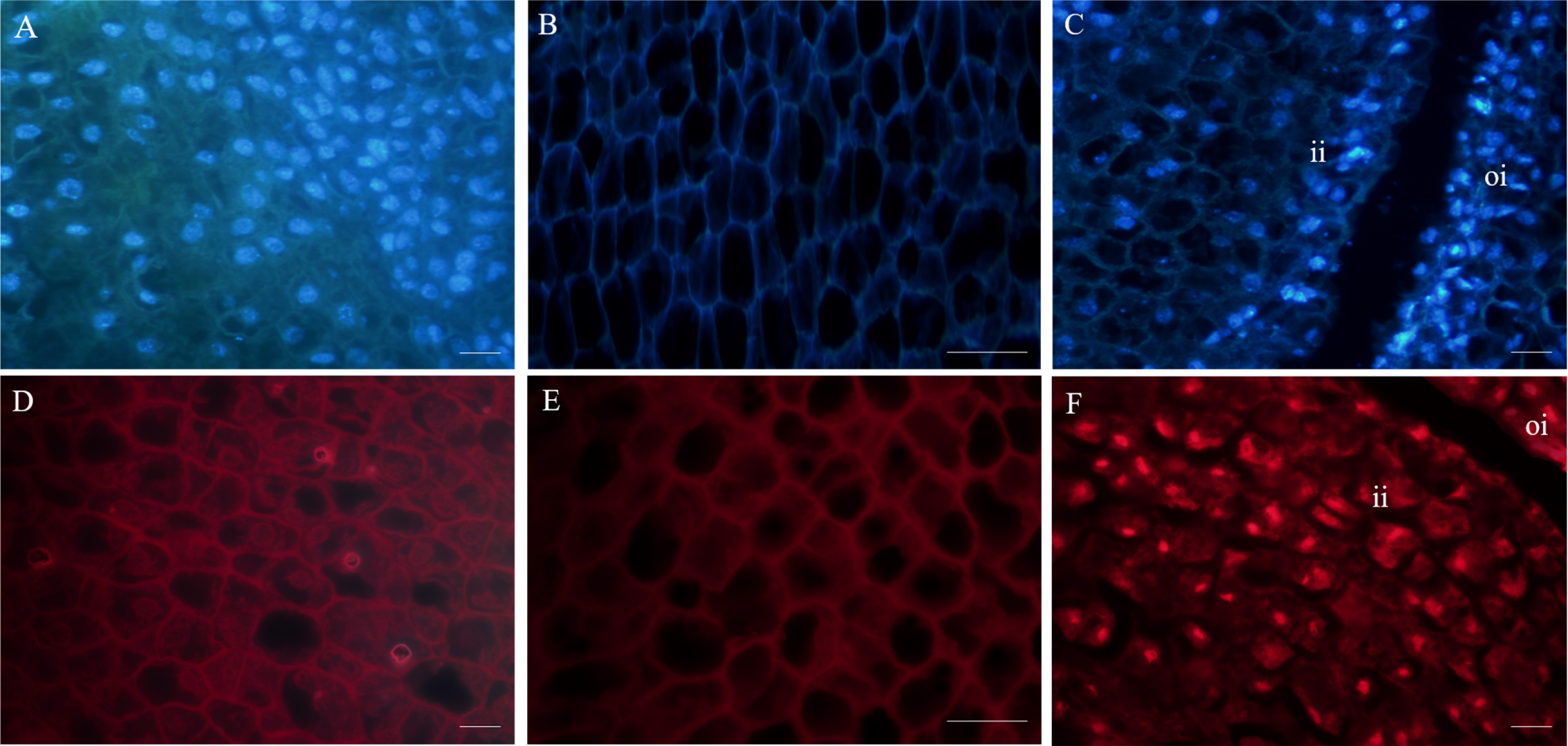
Fluorescent labeling of the nuclei of fertile and abortive ovules. (A, D) Fluorescent labeling of cell nucleus of the outer integument of fertile ovules, the cells showed large nucleus with obvious nucleoli; (B, E) fluorescent labeling of the inner integument of fertile ovules, the nucleolus disappeared; (C, F) fluorescent labeling of the outer integument of abortive ovules, the nucleus shows irregular appearance, “oi” represents outer integument and “ii” represents inner integument; Bar = 20 μm.

Fluorescence microscopy revealed that in the outer integument of fertile ovules, cell nuclei were in the shape of regular sphere, and the nuclear membrane could be seen in a sharp outline. However, the fluorescence intensity of DAPI labelling was higher than that of PI (Figs. 3A, 3D). In the inner integument of fertile ovules, no nuclei were detected in the cells (Figs. 3B, 3E). In the abortive ovules, there was little difference in the DAPI and PI fluorescence intensity. Notably, nucleoli of the outer integument and inner integument all exhibited intense fluorescence, and it was worth noting that the cells of inner integument had obvious nucleus. In addition, the cells of the outer and inner integument were abnormally shaped and had a smaller size. Cell nucleoli were detected as bright fluorescent spots (Figs. 3C, 3F).

### Ultrastructure of fertile and abortive ovules

**Figure 4:**
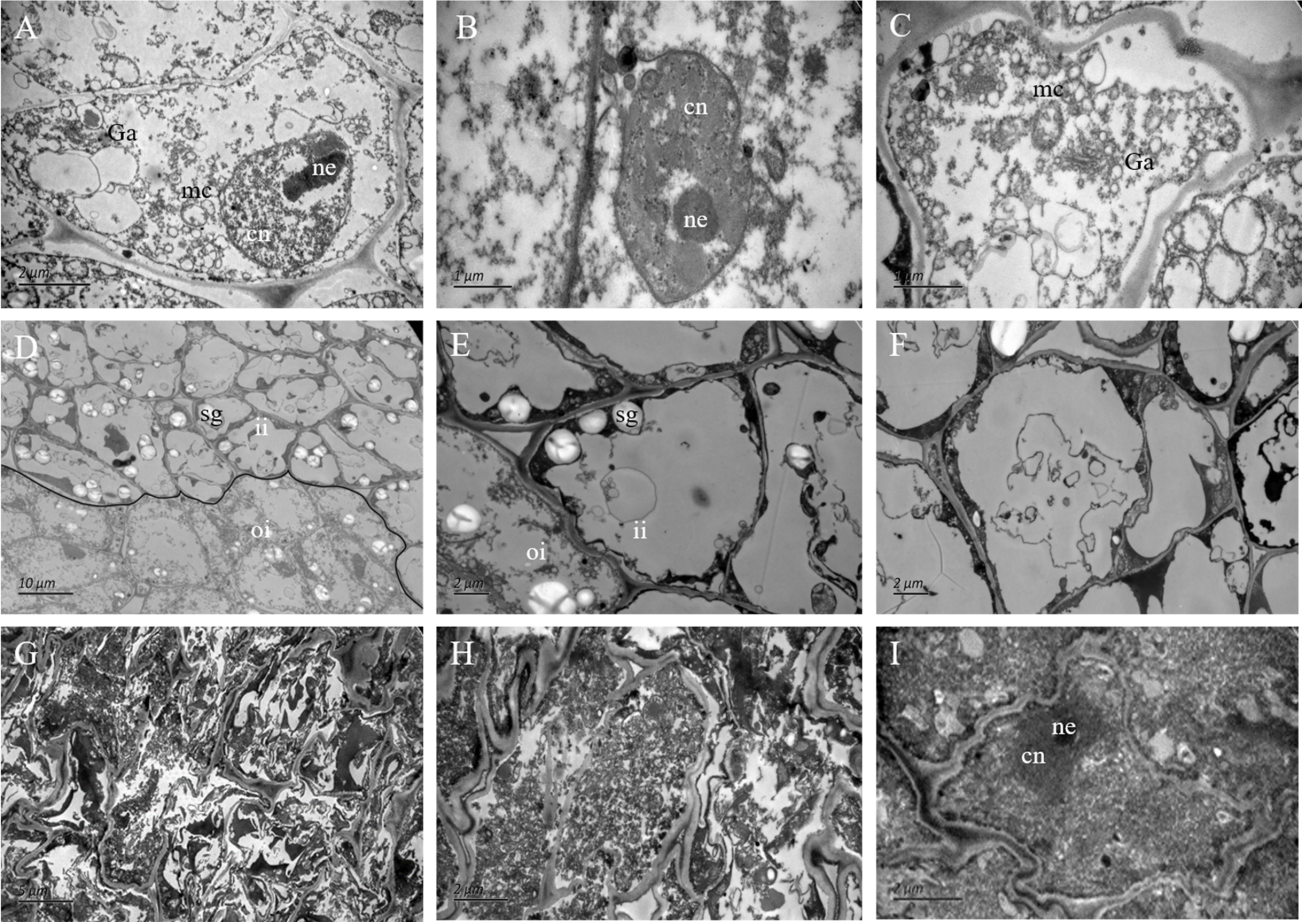
The ultrastructure observation of fertile and abortive ovules. (A–C) The ultrastructural observation of the outer integument of fertile ovules, cells had regular shapes and complete organelles; (D–F) the ultrastructural observation of the inner integument of fertile ovules, cell organelles and nucleus were degraded; (G–I) the ultrastructure observation of abortive ovules integument, a series of changes occurred in cells, such as the grave aggregation of cytoplasm and disintegration of cellular organelle; cn, cell nucleus; ne, nucleolus; mc, mitochondria; Ga, Golgi apparatus; sg, starch grain; ii, inner integument; oi, outer integument; The inner integument and the outer integument were distinguished by black line.

In the outer integument of fertile ovules, the cells were regularly shaped and contained a complete cell wall and plasma membrane. The cell nuclei were structurally intact and contained a clear and complete nuclear membrane and nucleoli. Meanwhile, mitochondria, Golgi apparatus, endoplasmic reticulum and other organelles all had complete structures, and several mitochondria were distributed around the cell membrane and nucleus (Figs. 4A–4C). Surprisingly, the ultrastructure features of the inner integument showed characteristics of apoptotic cells. There were distinct dividing lines between the inner and outer integuments (Fig. 4D). In the inner integument of fertile ovules, cell organelles were degraded, the cell contents were diminished, a cavity had formed inside the cells, and only starch grains were present in the cells (Figs. 4E, 4F). In the abortive ovules, the ultrastructure features of the outer integument showed an abnormal cell shape, irregular thickening of the cell walls, nuclear membrane rupture, substantial cytoplasm aggregation, and nuclear membrane damage. Furthermore, the organelles in these cells were difficult to distinguish (Figs. 4G–4I).

### Primary data analysis and protein identification in the fertile and abortive ovules

Proteins were extracted from fertile and abortive ovules. The total protein content extracted from fertile ovules was 536 µg at 1.79 µg/µL and that of abortive ovules was 220 µg at 0.73 µg/µL (Fig. S1A). The protein content of fertile ovules was 2.43-fold greater than that of abortive ovules. In addition, there were apparent differences between fertile ovules and abortive ovules in the patterns of whole-cell proteins. The experimental results showed that 25–66 KD proteins were in the greatest abundance, followed by 14–25 KD and 66–97 KD proteins. Most proteins of fertile ovules were concentrated in the 25–97 KD range, and most proteins of abortive ovules were concentrated in the 14–66 KD range (Fig. S1B). This showed that the molecular weights of proteins in the fertile ovules were higher than those in the abortive ovules. Proteomics technology was used to identify differentially expressed proteins (DEPs). A volcano plot of the DEPs is shown in Fig. S2A. We defined DEPs as those with a *P* value < 0.05 and with a fold-change >1.20 or <0.833 between fertile ovules and abortive ovules. The green and red areas represent significantly different DEPs, and the black areas represent non-significantly different DEPs. In total, 3,230 DEPs between fertile and abortive ovules were identified. Overall, 1,748 DEPs were upregulated and 1,482 DEPs were downregulated in the abortive ovules (Figs. S2A, S2B).

### Gene Ontology (GO) classification diagram and pathway enrichment of DEPs

GO annotation of the fertile and abortive ovules was performed using Blast2GO to identify the significantly enriched GO functional groups of the DEPs. The DEPs were grouped into three categories: molecular functions, cellular components and biological processes. For the molecular functions, the DEPs were predominantly distributed to the following categories: oxidoreductase, cationic binding enzyme, haem binding enzyme, antioxidant and calcium binding enzyme (Fig. S3). For the cellular components, the DEPs were predominantly distributed to the mitochondrial membrane system, respiratory chain reaction system, thylakoid, cell wall, proton transport ATP synthase complex and protease complex categories (Fig. S3). For the biological processes, the DEPs were predominantly distributed to the embryogenesis, hormone response, reactive oxygen metabolites, light intensity response, protein folding and cell death categories (Fig. S3). Significantly enriched KEGG pathways were determined based on a comparison of fertile and abortive ovules. DEPs were classified according to 17 KEGG categories. Among them, the pathways with more enriched differential proteins were amino acid synthesis and metabolism, biosynthesis of secondary metabolites, carbon fixation and carbon metabolism, and protein synthesis and hydrolysis (Fig. S4). We found that many pathways were involved in energy synthesis, such as glycolysis, pyruvate metabolism, oxidative phosphorylation and the TCA cycle. This indicated that energy synthesis was hindered in abortive ovules (Fig. S4). In addition, a number of DEPs were related to starch and sucrose metabolism, fatty acid synthesis and metabolism, purine and pyrimidine metabolism, the proteasome, and ascorbate and aldarate metabolism (Fig. S4).

### Functional categories of DEPs and detection of GAPDH expression in the fertile and abortive ovules

#### The abnormal biosynthesis of protein in the abortive ovules

**Figure 5:**
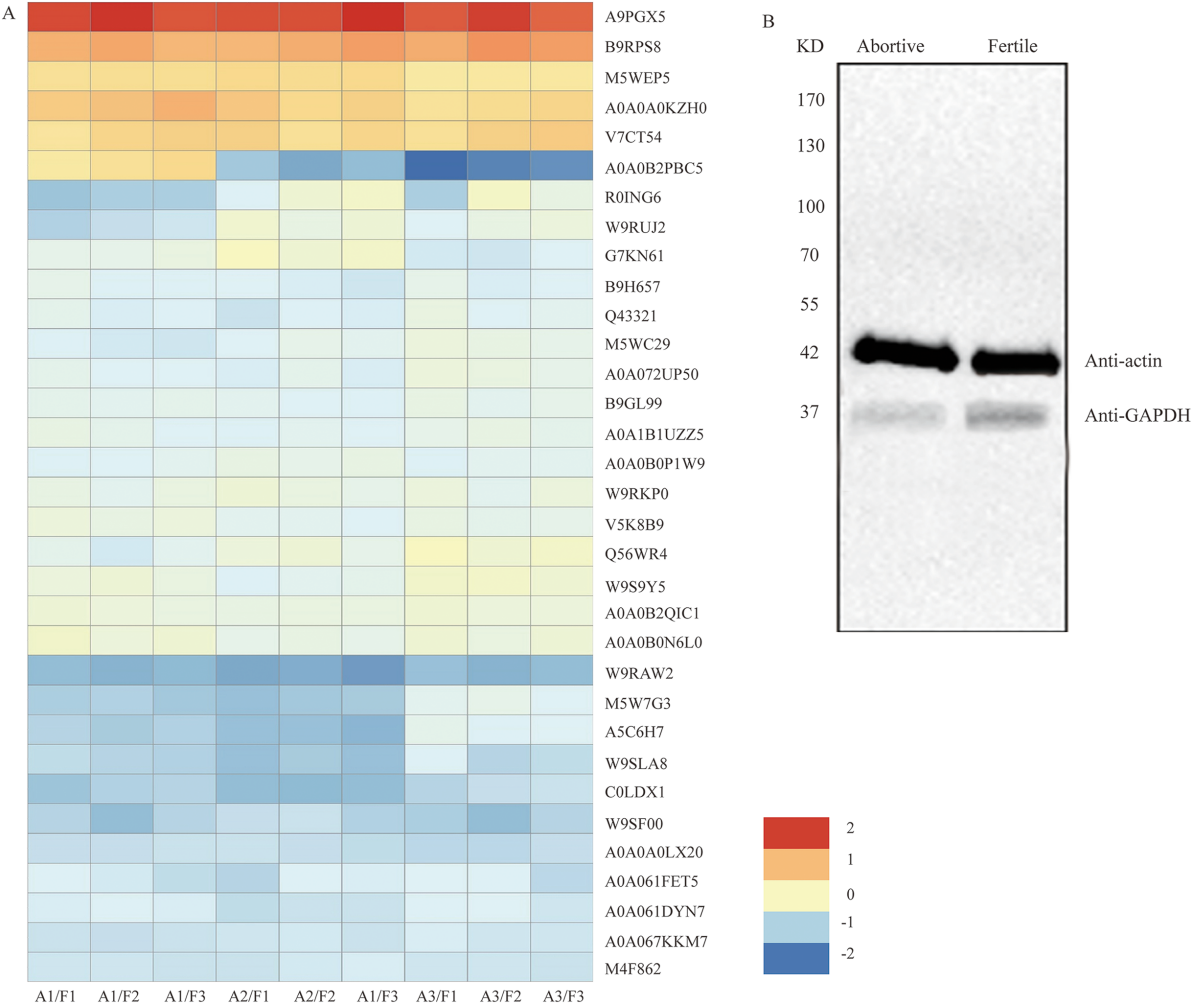
The clustering analysis of the 33 DEPs and detection of GAPDH expression in fertile ovules and abortive ovules. (A) These DEPs played out in protein synthesis, amino acid metabolism, energy synthesis and so on. “A” represents abortive ovules and “F” represents fertile ovules. (B) The protein gel blot probed with GAPDH antiserum detected a 37 kD band corresponding to the predicted size of GAPDH in the fertile ovules and abortive ovules. “A” represents abortive ovules and “F” represents fertile ovules.

Seven DEPs were identified as being involved in the synthesis and folding of proteins. Four translation initiation factors (Q56WR4, W9S9Y5, A0A0B2PBC5 and M5W7G3) were downregulated in abortive ovules (Table 1, Fig. 5A), which indicated that transcription and translation were likely to be suppressed, further decreasing protein synthesis. Protein disulfide isomerase (PDI) is an essential folding catalyst and chaperone of the endoplasmic reticulum, what’s more, it could catalyze disulfide bond formation, reduction, or isomerization, all of which are important for the maturation and proper folding of secreted or plasma membrane proteins (Urade, 2019; Xia et al., 2018; Wilkinson & Gilbert, 2004; Cho et al., 2011). Three PDIs (W9RKP0, A0A067KKM7 and R0ING6) were downregulated in abortive ovules (Table 1, Fig. 5A); therefore, the formation of disulfide bonds would be inhibited during protein synthesis, and misfolded proteins would not be identified and corrected.

**Table 1:** Down-regulated expression of the translation initiation factor in the abortive ovules.

Protein ID	Annotation	Expression ratio (A/F) ± SD	*P* value
Q56WR4	Translation initiation factor IF-2	0.7968 ± 0.0215	0.0001
W9S9Y5	Eukaryotic translation initiation factor 3 subunit B	0.7902 ± 0.0732	0.0017
A0A0B2PBC5	Eukaryotic initiation factor 4A-15	0.7317 ± 0.0409	0.0083
M5W7G3	Eukaryotic translation initiation factor 3 subunit B	0.5327 ± 0.0815	0.0001
W9RKP0	Protein disulfide-isomerase	0.7478 ± 0.0304	0.0302
A0A067KKM7	Protein disulfide-isomerase	0.6962 ± 0.0261	0.0001
R0ING6	Protein disulfide-isomerase	0.6896 ± 0.0534	0.0176

**Note:**

“A” represents abortive ovules and “F” represents fertile ovules; *P* ≤ 0. 05.

#### Abnormal amino acid metabolism in abortive ovules

Proteins comprise long chains of building blocks called amino acids, and each protein contains a specific number and arrangement of these molecules. Here, we found that amino acid metabolism and enzyme activity have an important effect on ovule fertility during ovule development. Nine DEPs were identified as being involved in the synthesis and metabolism of amino acids and were downregulated in abortive ovules. These DEPs were tryptophan synthase, peptidyl-prolyl cis-trans isomerase, aminotran_1_2 domain-containing protein, serine hydroxy methyltransferase, glutamate dehydrogenase, phosphoserine aminotransferase, eukaryotic aspartyl protease family protein, cysteine synthase and S-adenosylmethionine synthase (Table 2, Fig. 5A). The downregulation of tryptophan synthase, cysteine synthase and S-adenosylmethionine synthase would inhibit the synthesis of these three amino acids. The downregulation of peptidyl-prolyl cis-trans isomerase, serine hydroxy methyltransferase, glutamate dehydrogenase and phosphoserine aminotransferase would lead to the formation of proteins with abnormal structures.

**Table 2:** Differential expression of amino acid metabolism-related proteins.

Protein ID	Annotation	Expression ratio (A/F) ± SD	*P* value
A0A0B2QIC1	Tryptophan synthase	0.7805 ± 0.0513	0.0041
A0A0B0N6L0	Peptidyl-prolyl cis-trans isomerase	0.7789 ± 0.0326	0.0002
M5WXC0	Aminotran_1_2 domain-containing protein	0.7122 ± 0.0409	0.0114
W9RUJ2	Serine hydroxy methyltransferase	0.6983 ± 0.0139	0.0349
A0A072UP50	Glutamate dehydrogenase	0.6775 ± 0.0437	0.0008
B9H657	Phosphoserine aminotransferase	0.6458 ± 0.0652	0.0017
A0A061FET5	Eukaryotic aspartyl protease family protein	0.5581 ± 0.0287	0.0001
W9SLA8	Cysteine synthase	0.5095 ± 0.0461	0.0001
W9RAW2	S-adenosylmethionine synthase	0.3898 ± 0.0342	0.0335

**Note:**

“A” represents abortive ovules and “F” represents fertile ovules; *P* ≤ 0. 05.

#### Differential expression of peroxidases in the fertile and abortive ovules

Peroxidases are a family of oxidation-reduction enzymes that catalyse the oxidation of a series of organic compounds and are strongly associated with the activation of oxygen. Our analysis showed that six peroxidases (A9PGX5, B9RPS8, A0A0B0PJM7, A0A0A0KZH0, V7CT54 and M5WEP5) were upregulated (Table 3, Fig. 5A), which indicated that excessive reactive oxygen was produced in the abortive ovules.

**Table 3:** The differential expression of peroxidase.

Protein ID	Annotation	Expression ratio (A/F) ± SD	*P* value
A9PGX5	Peroxidase	4.0339 ± 0.0278	0.0009
B9RPS8	Peroxidase	2.4002 ± 0.0412	0.0004
A0A0B0PJM7	Peroxidase	2.387 ± 0.0125	0.0055
A0A0A0KZH0	Peroxidase	1.8988 ± 0.0326	0.0001
V7CT54	Peroxidase	1.8627 ± 0.0302	0.0007
M5WEP5	Peroxidase	1.7467 ± 0.0127	0.0008

**Note:**

“A” represents abortive ovules and “F” represents fertile ovules; *P* ≤ 0. 05.

#### Abnormal metabolism of the glycolytic pathway in abortive ovules

The glycolytic pathway is an important carbohydrate metabolism process in which ATP and pyruvate are produced. Seven DEPs were identified as being involved in the glycolytic pathway and were downregulated in abortive ovules. These were fructose-bisphosphate aldolase, 6-phosphogluconate dehydrogenase, decarboxylating enolase, pyruvate kinase, UDP-glucose 6-dehydrogenase, phosphoenolpyruvate carboxykinase and glyceraldehyde-3-phosphate dehydrogenase (Table 4, Fig. 5A). The downregulated expression of these enzymes would lead to reduced ATP and pyruvate synthesis, further impacting the TCA cycle and pyruvate metabolism. We analyzed the seven DEPs further from Table 4, the expression of GAPDH was the most notable. By looking up a large amount of literatures, we found this protein was a key enzyme in glycolysis, gluconeogenesis and the Calvin cycle, and which played a very important part in regulating mRNA formation, tRNA translocation, DNA replication, cell apoptosis and so on (Singh & Green, 1993; Krynetski et al., 2003; Hara et al., 2005). And so GAPDH was further researched in this subject.

**Table 4:** Differential expression of related enzymes in the glycolysis pathway.

Protein ID	Annotation	Expression ratio (A/F) ± SD	*P* value
W9QQH1	Fructose-bisphosphate aldolase	0.7116 ± 0.0251	0.0003
B9GL99	6-phosphogluconate dehydrogenase, decarboxylating	0.7127 ± 0.0731	0.0003
Q43321	Enolase	0.6883 ± 0.0197	0.0001
A0A067EZ22	Pyruvate kinase	0.6845 ± 0.0276	0.0006
M5WC29	UDP-glucose 6-dehydrogenase	0.6791 ± 0.0305	0.0168
A0A1B1UZZ5	Phosphoenolpyruvate carboxykinase	0.5999 ± 0.0751	0.0001
C0LDX1	Glyceraldehyde-3-phosphate dehydrogenase	0.4714 ± 0.0264	0.0335

**Note:**

“A” represents abortive ovules and “F” represents fertile ovules; *P* ≤ 0. 05.

#### Western blot analysis of glyceraldehyde-3-phosphate dehydrogenase (GAPDH)

Among the differentially expressed proteins that were enriched in the glycolytic pathway, the expression ratio of GAPDH (C0LDX1) was the lowest (0.4714), that is, based on the results of iTRAQ analysis, the expression of GAPDH showed the most obvious difference between fertile ovules and abortive ovules. As we all know, GAPDH is a key enzyme in glycolysis and an important enzyme in organisms. And the predicated protein product of GAPDH comprises 336 amino acids. In the differentially expressed proteins that were enriched in the glycolytic pathway, the expression ratio of GAPDH (C0LDX1) was the lowest (0.4714). Then the expression of GAPDH in fertile ovules and abortive ovules was detected by western blot and was in line with previous findings. As shown, consistent with predicted sizes of mature GAPDH protein, specific band of 37 KD was detected in fertile ovules and abortive ovules protein extracts probed with GAPDH antisera. As expected, we found that the expression of GAPDH in fertile ovules was higher than in abortive ovules (Fig. 5B).

## Discussion

Fruit and seed set establish yield potential of chestnut, and hence these developmental events are at the heart of determining global nut yield. In the process of our study, the only fertile ovule could be distinguished in the ovary at 20 DAA, and the rate of ovule abortion reached 94%. The chestnut thorns around the burrs were hardened, and the volume of the chestnut burrs and ovaries markedly increased. We observed the anatomy of the ovaries and found that the position of the fertile ovules was unfixed. This indicated that the position was not a key aspect of ovule fertility. An analysis of the ovary microstructure suggested that there were two ovules of varying sizes in a locule, and larger ovules were more likely to develop into fertile ovules. In addition, the microstructures of abortive ovules and fertile ovules were considerably different. The ovule microstructure analysis indicated that the embryo developed into a globular form in fertile ovules. By contrast, in the abortive ovules the embryo sac was not properly formed, which indicated that double fertilization could not be accomplished in abortive ovules. Compared with fertile ovules, there was a large gap between the inner and outer integuments of abortive ovules. This observation was in accordance with the findings of Wetzsteini et al. (2013).

Vertical sections of the fertile ovules showed no gap between the inner and outer integuments. The fluorescence labelling and ultrastructure observations indicated a substantial difference between the fertile and abortive ovules, we speculate that this phenomenon was caused by the breakage of the nuclear membrane. In the outer integument of fertile ovules, the cells had regular nuclei and abundant organelles, especially mitochondria, which indicated that they had robust metabolism (Souza, Wang & Dehesh, 2017). However, in the inner integument of fertile ovules, cell organelles and nuclei had degraded, which led to an empty cell cavity, indicating that these cells had died at a much earlier stage. For abortive ovules, typical ultrastructures of apoptotic cells were observed by electron microscopy, such as abnormally shaped cells, irregular thickening of the cell walls, and rupture of nuclear membranes which were similar to previous researches (Li et al., 2018; Ramírez-Sánchez et al., 2018). However, whether ovule abortion is caused by programmed cell death requires further research.

It is well known that the development of ovules is an intensive energy-demanding process. Our analysis identified 7 DEPs that were involved in various cellular processes such as protein synthesis and folding (Yuen, Matsumoto & Christopher, 2013; Matsusaki et al., 2016). These downregulated proteins would inhibit mRNA translation and cause the accumulation of misfolded proteins. Moreover, the downregulation of 9 DEPs involved in amino acid synthesis and metabolism would further inhibit protein synthesis and result in decreased free amino acids in abortive ovules. This would increase the osmotic potential of cells, reduce their ability to absorb water, and inhibit their growth and development.

Peroxidase (POD) is ubiquitous in plants and is closely related to various important physiological activities (Henriksen et al., 2001; Hoffmann et al., 2020). It is a key enzyme in the plant antioxidant system, removing excess reactive oxygen from the plant (Kimura & Kawano, 2015; Guan et al., 2017; Liebthal, Maynard & Dietz, 2018). When plant tissues or cells are in an adverse growth environment, a large amount of reactive oxygen species (ROS) accumulate. The harmful impact of ROS on normal metabolic processes leads to pathologic changes because ROS interacts with various biological compounds inside and outside of cells (Baxter, Mittler & Suzuki, 2014; Chapman et al., 2019). In the abortive ovules, the activity of POD was significantly upregulated, which indicated that ROS was abnormally metabolized and accumulated. This would increase the senescence and lipid peroxidation of abortive ovules, causing further injury and cell death. This finding was consistent with those of previous reports on the causes of ovule abortion in other plants (Wang, Zhang & Allen, 1999; Sun, Cui & Hayser, 2005; Hauser et al., 2006).

The development of ovules is an intensive energy-demanding process, but in the present study, the iTRAQ results showed that most of the DEPs were concentrated in the mitochondrial membrane system, the respiratory chain reaction system, and the proton transport ATP synthase complex, indicating that the respiratory system of abortive ovules is significantly different from that of fertile ovules. Moreover, the ultrastructural observations showed that the number of mitochondria in abortive ovule cells was much lower than that in fertile ovule cells. As mentioned above, the occurrence of ovule abortion was closely related to the number and function of mitochondria. In addition, the intensity of material and energy metabolism directly reflects the growth and development of ovules, and the glycolysis reactions provide the energy to maintain the life of the cell (Ireland & Dennis, 1981; Wakao, Andre & Benning, 2007). This study identified that eight DEPs were involved in the glycolysis pathway in key roles; these DEPs were downregulated in the abortive ovules. The downregulation of enzymes related to glycolytic metabolism would ultimately result in a decreased amount of pyruvate in the citric acid cycle, while it could also give rise to reduced ATP synthesis. In addition, GAPDH is considered as a critical enzyme in plants and plays an important role in glycolysis and gluconeogenesis. The iTRAQ and western blot results showed that GAPDH was significantly downregulated in the abortive ovules. This would disrupt the level of 1,3-bisphosphoglycerate, leading to abnormal glycolysis, even abnormality of multiple biological pathways, such as mRNA formation, DNA replication, scavenging of free radical and so on. In these biological pathways, insufficient energy supply and the excessive free radical might be the main cause of the ovule abortion.

## Conclusions

According to cytological studies, we found significant differences in the microstructures and ultrastructures of fertile and abortive ovules. Meanwhile, iTRAQ analysis showed that screened DEPs in the fertile ovules and abortive ovules were largely enriched in protein synthesis, accumulation of active oxygen free radical, energy synthesis and so on. To sum up, our findings provide new insights into the mechanisms responsible for ovule abortion and will promote further studies of Chinese chestnut.

## Supplemental Information

10.7717/peerj.11756/supp-1 Supplemental Information 1 Supplementary Figures.

## Additional Information and Declarations

### Data Availability

The following information was supplied regarding data availability: The mass spectrometry proteomics data are available at the ProteomeXchange Consortium via the PRIDE partner repository: PXD021317.

## References

[ref-1] Awasthi A, Paul P, Kumar S, Verma SK, Prasad R, Dhaliwal HS (2012). Abnormal endosperm development causes female sterility in rice insertional mutant *OsAPC6*. Plant Science.

[ref-2] Baxter A, Mittler R, Suzuki N (2014). ROS as key players in plant stress signalling. Journal of Experimental Botany.

[ref-3] Cagnola JL, Ibarra SE, Chimenti C, Ricardi MM, Delzer B, Ghiglione H, Zhu T, Otegui ME, Estevez JM, Casal JJ (2018). Reduced expression of selected FASCICLIN-LIKE ARABINOGALACTAN PROTEIN genes associates with the abortion of kernels in field crops of *Zea mays* (maize) and of Arabidopsis seeds. Plant Cell & Environment.

[ref-4] Chang XX, Liu FY, Lin ZX, Qiu JS, Peng C, Lu YS, Guo XB (2020). Phytochemical profiles and cellular antioxidant activities in chestnut (*Castanea mollissima* BL.) kernels of five different cultivars. Molecules.

[ref-5] Chapman JM, Muhlemann JK, Gayomba SR, Muday GK (2019). RBOH-dependent ROS synthesis and ROS scavenging by plant specialized metabolites to modulate plant development and stress responses. Chemical Research in Toxicology.

[ref-6] Chen GS, Li JT, Liu Y, Zhang Q, Gao YR, Fang KF, Cao QQ, Qin L, Xing Y (2019). Roles of the GA-mediated SPL gene family and miR156 in the floral development of Chinese chestnut (*Castanea mollissima*). International Journal of Molecular Sciences.

[ref-7] Cheng PY, Li HJ, Yuan LL, Li HY, Xi LL, Zhang JJ, Liu J, Wang YD, Zhao HP, Zhao HX, Han SH (2018). The ERA-related GTPase AtERG2 associated with mitochondria 18S RNA is essential for early embryo development in Arabidopsis. Frontiers of Plant Science.

[ref-8] Cho EJ, Yuen CYL, Kang BH, Ondzighi CA, Staehelin LA, Christopher DA (2011). Protein disulfide isomerase-2 of Arabidopsis mediates protein folding and localizes to both the secretory pathway and nucleus, where it interacts with maternal effect embryo arrest factor. Molecules and Cells.

[ref-9] Du BS, Zhang Q, Cao QQ, Xing Y, Qin L, Fang KF (2020). Changes of cell wall components during embryogenesis of *Castanea mollissima*. Journal of Plant Research.

[ref-10] Geng XX, Ye JL, Yang XT, Li S, Zhang LL, Song XY (2018). Identification of proteins involved in carbohydrate metabolism and energy metabolism pathways and their regulation of cytoplasmic male sterility in wheat. International Journal of Molecular Sciences.

[ref-11] Guan TL, Shen JH, Fa Y, Su SY, Wang X, Li HM (2017). Resistance-breaking population of meloidogyne incognita utilizes plant peroxidase to scavenge reactive oxygen species, thereby promoting parasitism on tomato carrying Mi-1 gene. Biochemical and Biophysical Research Communications.

[ref-12] Hamamura Y, Saito C, Awai C, Kurihara D, Miyawaki A, Nakagawa T (2011). Live-cell imaging reveals the dynamics of two sperm cells during double fertilization in *Arabidopsis thaliana*. Current Biology.

[ref-13] Hara MR, Agrawal N, Kim SF, Cascio1 MB, Fujimuro M, Ozeki Y, Takahashi M, Cheah JH, Tankou1 SK, Hester LD, Ferris CD, Hayward SD, Snyder SH, Sawa A (2005). S-nitrosylated GAPDH initiates apoptotic cell death by nuclear translocation following Siah1 binding. Nature Cell Biology.

[ref-14] Hauser BA, Sun K, Oppenheimer DG, Sage TM (2006). Changes in mitochondrial membrane potential and accumulation of reactive oxygen species precede ultrastructural changes during ovule abortion. Planta.

[ref-15] Henriksen A, Mirza O, Teilum K, Smulevich G, Welinder KG, Gajhede M (2001). Structure of soybean seed coat peroxidase: a plant peroxidase with unusual stability and haem-apoprotein interactions. Protein Science.

[ref-16] Hoffmann N, Benske A, Betz H, Schuetz M, Samuels AL (2020). Laccases and peroxidases co-localize in lignified secondary cell walls throughout stem development. Plant Physiology.

[ref-17] Ireland RJ, Dennis DT (1981). Isoenzymes of the glycolytic and pentose phosphate pathways during the development of castor oil seed. Canadian Journal of Botany.

[ref-18] Jiang N, Liang LY, Tian CM (2020). Gnomoniopsis chinensis (*Gnomoniaceae, Diaporthales*), a new fungus causing canker of Chinese chestnut in Hebei Province. China MycoKeys.

[ref-19] Kimura M, Kawano T (2015). Salicylic acid-induced superoxide generation catalyzed by plant peroxidase in hydrogen peroxide-independent manner. Plant Signaling and Behavior.

[ref-20] Krynetski EY, Krynetskaia NF, Bianchi ME, William EE (2003). Anuclear protein complex containing high mobility group proteins B1 and B2, heat shock cognate protein 70, ERp60, and glyceraldehyde-3-phosphate dehydrogenase is involved in the cytotoxic response to DNA modified by in-corporation of anticancer nucleoside analogues. Cancer Research.

[ref-21] Leshem Y, Johnson C, Wuest SE, Song X, Ngo QA, Grossniklaus U (2012). Molecular characterization of the glauce mutant: a central cell-specific function is required for double fertilization in arabidopsis. Plant Cell.

[ref-22] Li C, Li C, Wang BB, Zhang Q, Fu KY, Gale WJ, Li CY (2018). Programmed cell death in wheat (*Triticum aestivum* L.) endosperm cells is affected by drought stress. Protoplasma.

[ref-23] Liebthal M, Maynard D, Dietz KJ (2018). Peroxiredoxins and redox signaling in plants. Antioxidants and Redox Signaling.

[ref-24] Liu H, Liu YZ, Zheng SQ, Jiang JM, Wang P, Chen W (2010). Comparative proteomic analysis of longan (*Dimocarpus longan* Lour.) seed abortion. Planta.

[ref-25] Liu ZN, Yu XL, Wang FZ, Hu A, Liu YP, Lu G (2012). Physiological, biochemical, and molecular characterization of a new female sterile mutant in turnip. Plant Growth Regulation.

[ref-26] Matsusaki M, Okuda A, Masuda T, Koishihara K, Mita R, Iwasaki K, Hara K, Naruo Y, Hirose A, Tsuchi Y, Urade R (2016). Cooperative protein folding by two protein thiol disulfide oxidoreductases and ERO1 in Soybean. Plant Physiology.

[ref-27] Nie HS, Cheng C, Hua JP (2020). Mitochondrial proteomic analysis reveals that proteins relate to oxidoreductase activity play a central role in pollen fertility in cotton. Journal of Proteomics.

[ref-28] Qin J, Zhang J, Liu D, Yin C, Wang F, Chen P, Chen H, Ma J, Zhang B, Xu J, Zhang M (2016). iTRAQ-based analysis of developmental dynamics in the soybean leaf proteome reveals pathways associated with leaf photosynthetic rate. Molecular Genetics and Genomics.

[ref-29] Ramírez-Sánchez M, Huber DJ, Vallejos CE, Kelley K (2018). Physiological, molecular and ultrastructural analyses during ripening and over-ripening of banana (*Musa* spp., AAA group, Cavendish sub-group) fruit suggest characteristics of programmed cell death. Journal of the Science of Food and Agriculture.

[ref-30] Robinson-Beers K, Pruitt RE, Gasser CS (1992). Ovule development in wild-type Arabidopsis and two female-sterile mutants. Plant Cell.

[ref-31] Singh R, Green MR (1993). Sequence-specific binding of transfer RNA by glyceraldehyde-3-phosphate dehydrogenase. Science.

[ref-32] Souza AD, Wang JZ, Dehesh K (2017). Retrograde signals: integrators of interorganellar communication and orchestrators of plant development. Annual Review of Plant Biology.

[ref-33] Sun K, Cui Y, Hayser BA (2005). Environmental stress alters genes expression and induces ovule abortion: reactive oxygen species appear as ovules commit to abort. Planta.

[ref-34] Sun HL, Shi T, Song J, Xu YS, Gao ZH, Song XX, Ni ZJ, Cai BH (2016). Pistil abortion in Japanese apricot (*Prunus mume* et Zucc.): isolation and functional analysis of *PmCCoAOMT* gene. Acta Physiologiae Plantarum.

[ref-35] Tang JJ, Kato M (2002). A comparatively histological observation on the megagametophytic abortion of female-sterile rice FS-1 and its maternal parent fujisaka. Acta Biologiae Experimentalis Sinica.

[ref-36] Urade R (2019). Oxidative protein folding in the plant endoplasmic reticulum. Bioscience Biotechnology and Biochemistry.

[ref-37] Wakao S, Andre C, Benning C (2007). Functional analyses of cytosolic glucose-6-phosphate dehydrogenases and their contribution to seed oil accumulation in Arabidopsis. Plant Physiology.

[ref-38] Wang J, Wang XR, Zhou Q, Yang JM, Guo HX, Yang LJ, Liu WQ (2016). iTRAQ protein profile analysis provides integrated insight into mechanisms of tolerance to TMV in tobacco (*Nicotiana tabacum*). Journal of Proteomics.

[ref-39] Wang J, Zhang H, Allen RD (1999). Overexpression of an Arabidopsis peroxisomal ascorbate peroxidase gene in tobacco increases protection against oxidative stress. Plant and Cell Physiology.

[ref-49] Wasinger VC, Cordwell SJ, Cerpa-Poljak A, Yan JX, Gooley AA, Wilkins MR, Duncan MW, Harris R, Williams KL, Humphery-Smith I (1995). Progress with gene-product mapping of the Mollicutes: Mycoplasma genitalium. Electrophoresis.

[ref-40] Wetzsteini HY, Yi WG, Porte JA, Ravid N (2013). Flower position and size impact ovule number per flower, fruitset, and fruit size in pomegranate. Journal of American Society for Horticultural Science.

[ref-41] Wilkins MR, Pasquali C, Appel RD, Ou K, Golaz O, Sanchez JC, Yan JX, Gooley AA, Hughes A, Humphery-Smith L, Williams KL, Hochstrasser DF (1996). From proteins to proteomes: large scale protein identification by two-dimensional electrophoresis and amino acid analysis. Biotechnology.

[ref-42] Wilkinson B, Gilbert HF (2004). Protein disulfide isomerase. Biochimica Et Biophysica Acta.

[ref-43] Xia KF, Zeng X, Jiao ZL, Li ML, Xu WJ, Nong QD, Mo H, Cheng TH, Zhang MY (2018). Formation of protein disulfide bonds catalyzed by ospdil1;1 is mediated by microrna5144-3p in rice. Plant and Cell Physiology.

[ref-44] Yang L, Wu Y, Yu M, Mao BG, Wang JB (2016). Genome-wide transcriptome analysis of female-sterile rice ovule shed light on its abortive mechanism. Planta.

[ref-45] Yuen CYA, Matsumoto KO, Christopher DA (2013). Variation in the subcellular localization and protein folding activity among *Arabidopsis thaliana* homologs of protein disulfide isomerase. Biomolecules.

[ref-46] Zhang M, Song XX, Lv K, Yao Y, Gong ZX, Zheng CX (2017). Differential proteomic analysis revealing the ovule abortion in the female-sterile line of *Pinus tabulaeformis* Carr. Plant Science.

[ref-47] Zhang FJ, Wang ZQ, Dong W, Sun C, Wang HB, Song AP, He LZ, Fang WM, Chen FD, Teng NJ (2014). Transcriptomic and proteomic analysis reveals mechanisms of embryo abortion during chrysanthemum cross breeding. Scientific Reports.

[ref-48] Zhu YX, Li YD, Zang SL, Zhang X, Yao JW, Luo Q, Sun F, Wang X (2019). Genome-wide identification and expression analysis reveal the potential function of ERF (Ethylene responsive factor) gene family in response to Botrytis cinerea infection and ovule development in grapes (*Vitis vinifera* L.). Plant Biology.

