# Supplementary material for: Morphological observation and protein expression of fertile and abortive ovules in Castanea mollissima
Source: PeerJ. 2021 Jul 21;9:e11756. doi: 10.7717/peerj.11756 (PMC8308611; doi:10.7717/peerj.11756)

Supplementary data

Morphological observation and protein expression of fertile and abortive ovules in *Castanea mollissima*

Bingshuai Du[1,2], Qing Zhang[3], Qingqin Cao[1,3], Yu Xing[1,3], Ling Qin[1,3,*] & Kefeng Fang[1,2,4,*]

[1] Beijing Advanced Innovation Center for Tree Breeding by Molecular Design, Beijing University of Agriculture, 102206, China;

[2] College of Landscape Architecture, Beijing University of Agriculture, Beijing 102206, China;

[3] Key Laboratory for Agricultural Application and New Technique, College of Plant Science and Technology, Beijing University of Agriculture, Beijing 102206, China.

[4] Key Laboratory of Urban Agriculture (North China, Ministry of Agriculture P. R. China), Beijing University of Agriculture, Beijing 102206, China;

[*] Corresponding: fangkefeng@126.com (Kefeng Fang); qinlingbac@126.com (Ling Qin)

Supplementary Figures

Supplementary Figure 1: Quantitative analysis of protein in fertilized and abortive ovules. (A) The results of protein extraction from fertilized and abortive ovules; (B) The SDS-PAGE electropherogram of total protein of fertilized and abortive ovules. F, fertilized ovules; A, abortive ovules. “a” and “b” showed that there is a significant difference between fertile ovules and abortive ovules.

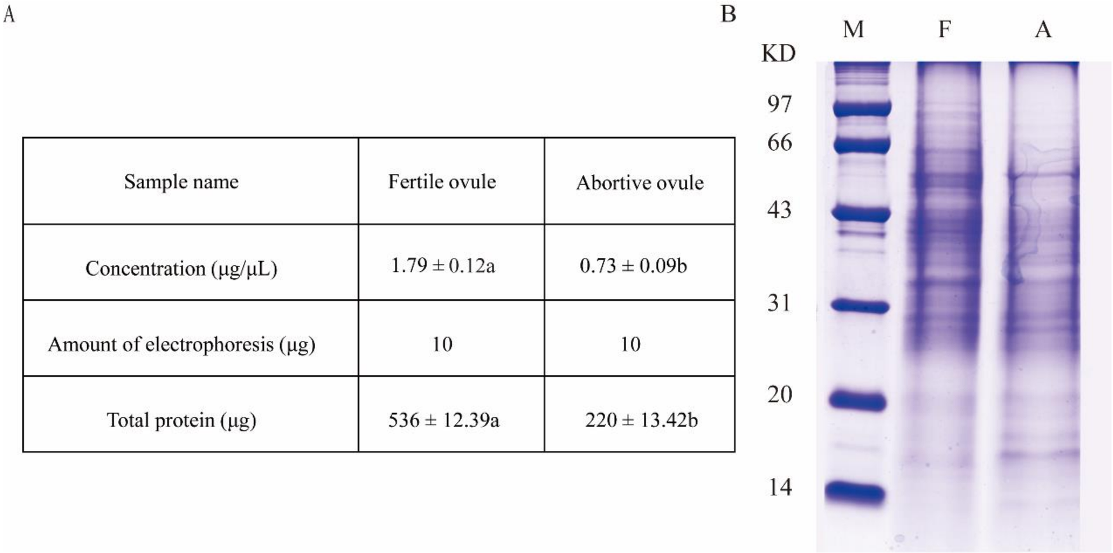
A

Sample name	Fertile ovule	Abortive ovule
Concentration (μg/μL)	1.79 ± 0.12a	0.73 ± 0.09b
Amount of electrophoresis (μg)	10	10
Total protein (μg)	536 ± 12.39a	220 ± 13.42b

Supplementary Figure 2: Separation of differentially expressed proteins in fertile and abortive ovules. (A) The volcanic map of DEPs; (B) The number of DEPs; “A” represents upregulated DEPs, “B” represents downregulated DEPs, “C” shows no significant DEPs, “FC” represents fold change; “up” shows upregulated DEPs, “down” shows downregulated DEPs.

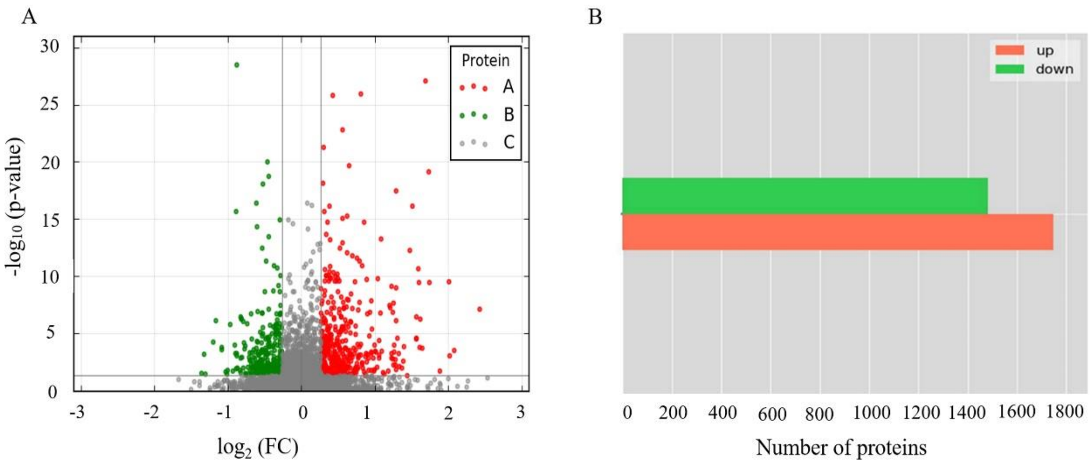

Supplementary Figure 3: GO classifications of DAPs in fertile ovules and abortive ovules. The DEPs were grouped into three categories: molecular functions, cellular components and biological processes. The x-axis represents the number of enriched DEP in the main category and the y-axis represents each GO term.

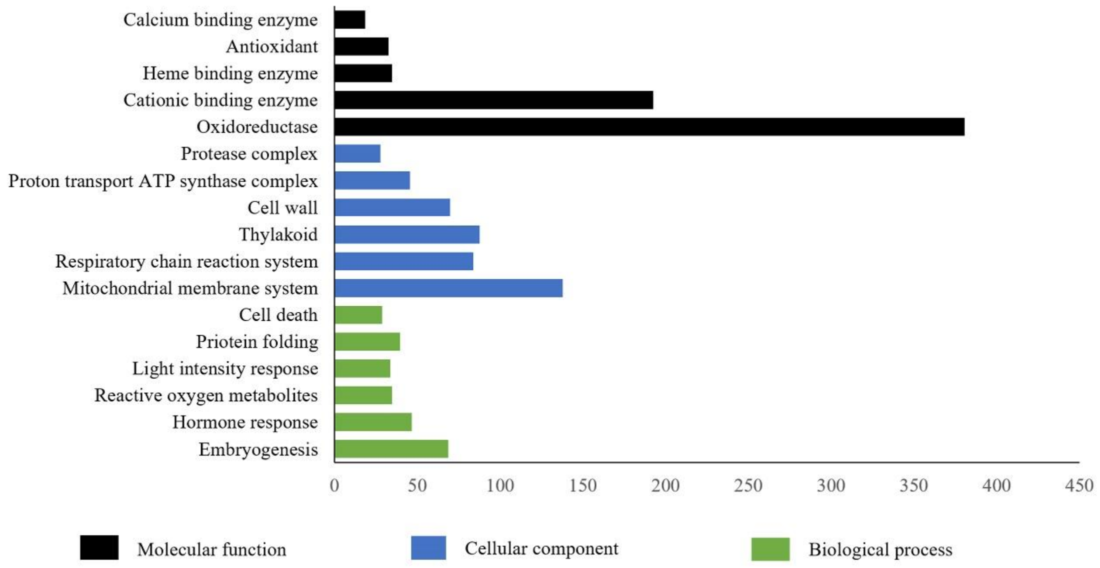

Supplementary Figure 4: KEGG pathway enrichment analyses of fertile and abortive ovules. These DEPs were mainly enriched in 17 biological pathways. The vertical axis shows the annotations of the KEGG metabolic pathways. The horizontal axis represents the DEPs numbers annotated in each pathway.

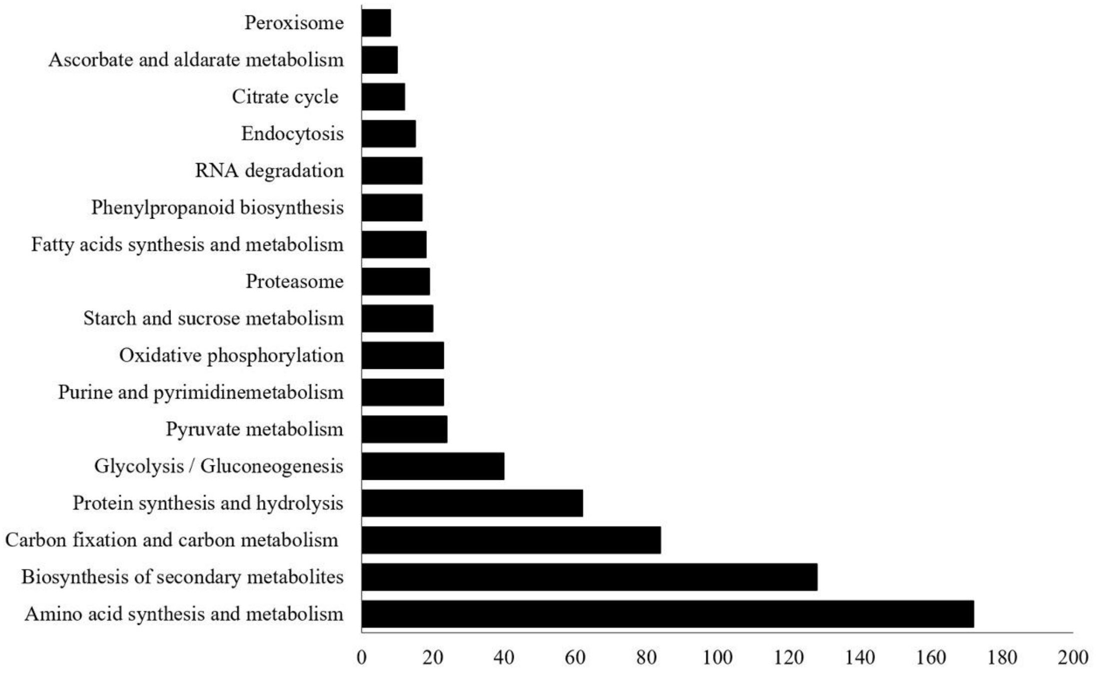

Supplementary Figure 5: Full blots: (A) The SDS-PAGE electropherogram of total protein of fertile and abortive ovules. “M” represents marker, “F” represents fertile ovules and “A” represents abortive ovules. (B) Detection of GAPDH expression in fertile ovules and abortive ovules. “A” represents abortive ovules and “F” represents fertile ovules.

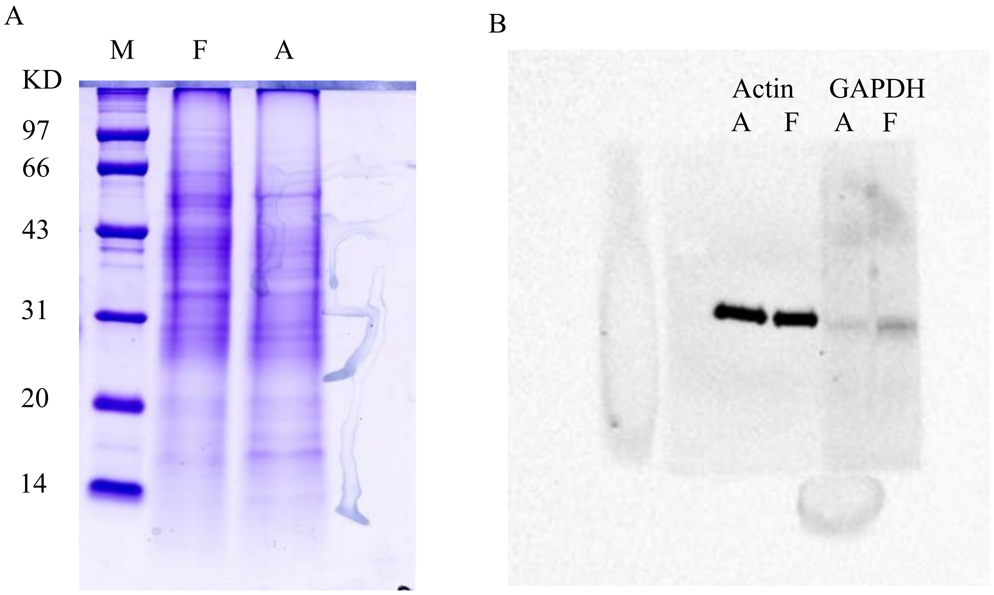

Supplement: Supplemental Information 1 [file peerj-09-11756-s001.pdf]
